# Highlighting uncertainty in clinical risk prediction using a model of emergency laparotomy mortality risk

**DOI:** 10.1038/s41746-022-00616-7

**Published:** 2022-06-08

**Authors:** Jakob F. Mathiszig-Lee, Finneas J. R. Catling, S. Ramani Moonesinghe, Stephen J. Brett

**Affiliations:** 1 Department of Surgery and Cancer, Imperial College London, London, UK; 2 Department of Anaesthesia and Perioperative Medicine, Royal Marsden Hospital, London, UK; 3 Surgical Outcomes Research Centre, Centre for Perioperative Medicine, Department for Targeted Intervention, University College London, London, UK; 4 University College London Hospitals National Institute for Health Research Biomedical Research Centre, London, UK; 5 Present Address: Imperial College London, The Commonwealth Building, The Hammersmith Hospital, Du Cane Road, London, W12 0NN UK; 6 Present Address: Imperial College London, St. Mary’s Campus, Norfolk Place, London, W2 1PG UK; 7 Present Address: University College London, Charles Bell House, 43-47 Foley Street, London, W1W 7TS UK; 8 Present Address: ICU West, The Hammersmith Hospital, Du Cane Road, London, W12 0HS UK

**Keywords:** Translational research, Risk factors, Prognosis, Statistics, Gastrointestinal diseases

## Abstract

Clinical prediction models typically make point estimates of risk. However, values of key variables are often missing during model development or at prediction time, meaning that the point estimates mask significant uncertainty and can lead to over-confident decision making. We present a model of mortality risk in emergency laparotomy which instead presents a distribution of predicted risks, highlighting the uncertainty over the risk of death with an intuitive visualisation. We developed and validated our model using data from 127134 emergency laparotomies from patients in England and Wales during 2013–2019. We captured the uncertainty arising from missing data using multiple imputation, allowing prospective, patient-specific imputation for variables that were frequently missing. Prospective imputation allows early prognostication in patients where these variables are not yet measured, accounting for the additional uncertainty this induces. Our model showed good discrimination and calibration (95% confidence intervals: Brier score 0.071–0.078, C statistic 0.859–0.873, calibration error 0.031–0.059) on unseen data from 37 hospitals, consistently improving upon the current gold-standard model. The dispersion of the predicted risks varied significantly between patients and increased where prospective imputation occurred. We present a case study that illustrates the potential impact of uncertainty quantification on clinical decision making. Our model improves mortality risk prediction in emergency laparotomy and has the potential to inform decision-makers and assist discussions with patients and their families. Our analysis code was robustly developed and is publicly available for easy replication of our study and adaptation to predicting other outcomes.

## Introduction

Numerous models are used to predict mortality in patients having general surgery^1^. These adjust their predictions based on some patient variables but do not include all potential determinants of mortality risk. A model that uses few variables relating to acute physiology, for example, makes predictions that average over different acute physiological states. Put more plainly, a patient with a predicted 10% risk of mortality has a set of characteristics (e.g. chronic health, planned surgical intervention) for which the population mortality is 10%. However, their individual risk might be higher or lower than this, based on factors which are not measured.

**Fig. 1:**
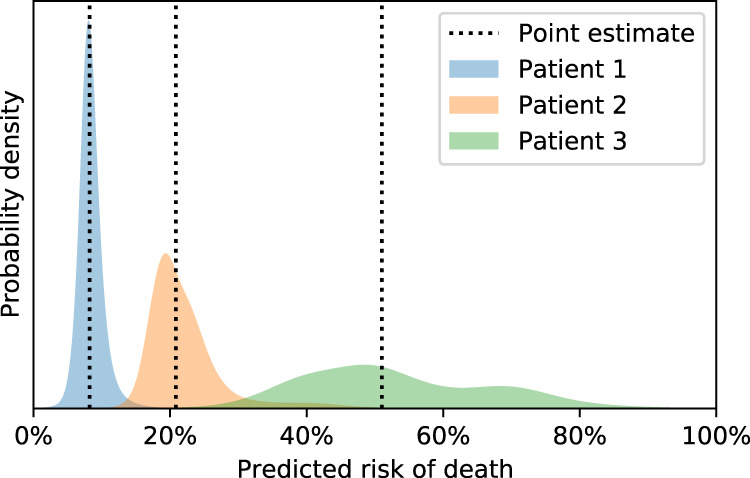
Point estimates of mortality risk contrasted with predicted distributions over mortality risk. The point estimates from conventional models mask whether our prediction is relatively certain (as in Patient 1) or uncertain (as in Patient 3). The median predicted risk is used as the point estimate in each case. Patients 2 and 3 may have a mortality risk much higher than the median, and the point estimates fail to communicate this.

When prediction models are used in the care of individual patients, their point estimates of risk (expected risk predictions) may mask significant uncertainty induced by the unmeasured variables^2^. If interpreted incorrectly, these point estimates have the potential to mislead patients and clinicians. It is for this reason that risk calculators stress the importance of clinical judgement alongside their use, or incorporate such judgement explicitly^3^. Figure 1 contrasts point estimates with *distributions* over mortality risk. Risk distributions make uncertainty obvious: the ‘spread’ (dispersion) of the distribution is commensurate with the uncertainty. A model that predicts such distributions could encourage cognisance of uncertainty and thus mitigate overconfident decision-making.

Emergency laparotomy (EL) is amongst the highest risk surgical procedures undertaken worldwide, with 30-day mortality ranging from 8–14% in developed healthcare systems^4–7^. The National Emergency Laparotomy Audit (NELA) was established in 2013 in the United Kingdom (UK), to record and improve perioperative care. Mortality risk estimation is an essential part of this effort, informing discussions with patients and carers, and helping to ensure senior involvement and Critical Care admission for those at higher risk. The NELA Calculator was developed for post hoc risk adjustment across cohorts from different hospitals but is increasingly used for patient-level mortality risk prediction in EL^8^. It is recognised in international guidelines^9^ and has been shown to have the greatest discriminatory power of the established surgical prediction models in EL cohorts from the UK^8,10^, Singapore^11^, Australia^12^ and New Zealand^13^.

This study aimed to develop and validate a model—referred to as ‘risk of death is uncertain in emergency laparotomy’ or RUNE—for preoperative use in EL, which predicts distributions over in-hospital mortality risk for individual patients. Whilst previous investigators have considered uncertain associations between individual risk factors and mortality in EL, to our knowledge none has propagated this uncertainty to the predictions of a multivariable mortality risk model. In particular, we sought to quantify the uncertainty arising from missing data, both during the model fitting process and when the model is used in practice. We also aimed for the model to capture important non-linear relationships between clinical variables and mortality, whilst ensuring that its inner workings are straightforward and easy to interpret. We compared RUNE’s performance with that of the NELA Calculator.

## Results

186 hospitals in England and Wales provided data from 127,148 cases, representing 81% of all ELs occurring in England and Wales during the study period^6^. Nine cases below 18 years of age and three cases above 109 years of age were excluded, leaving 127,134 cases for inclusion in our study. Before day 60 of postoperative care, 111,364 cases (87.6%) were discharged from the hospital alive and 14,343 (11.3%) had died in hospital. 1427 cases (1.1%) remained in the hospital on day 60.

Seven variables (sex, haemoglobin, C-reactive protein, surgical urgency, number of surgeries within 30 days, the severity of the intended surgery and predicted blood loss) were excluded from RUNE by backward elimination. Table 1 summarises RUNE’s remaining variables (including their missingness) for the study population, comparing cases across the first cross-validation split.

**Table 1:** Demographic information and RUNE covariates.

Variable^a^	Missing values^b^	Development cases^c^ (*n* = 102,713)	Validation cases^c^ (*n* = 23,389)
Age (years): median (IQR)	0 (0.0%)	67 (53–77)	67 (53–78)
Female^a^: *n* (%)	0 (0.0%)	53114 (51.7%)	12012 (51.4%)
Died: *n* (%)	0 (0.0%)	11587 (11.3%)	2640 (11.3%)
ASA physical status: median (IQR)	0 (0.0%)	3 (2–3)	3 (2–3)
Cardiovascular status: mode (*n*, %)	676 (0.5%)	No failure (74,686, 73.0%)	No failure (17,405, 74.4%)
Respiratory status: mode (*n*, %)	651 (0.5%)	No dyspnoea (74,212, 72.6%)	No dyspnoea (16,842, 72.0%)
Heart rate (beats min^−1^): median (IQR)	1392 (1.1%)	90 (79–102)	89 (78–102)
Non-sinus rhythm: *n* (%)	875 (0.7%)	19,503 (19.1%)	4664 (19.9%)
Systolic pressure (mmHg): median (IQR)	1731 (1.4%)	125 (110–140)	125 (110–140)
Sodium (mmol L^−1^): median (IQR)	353 (0.3%)	137 (134–140)	137 (134–140)
Potassium (mmol L^−1^): median (IQR)	673 (0.5%)	4.1 (3.8–4.5)	4.1 (3.7–4.5)
White cell count (×10^9^ L^−1^): median (IQR)	496 (0.4%)	11.1 (7.8–15.4)	11.1 (7.8–15.3)
Creatinine (mg/dL): median (IQR)	1908 (1.5%)	0.86 (0.69–1.15)	0.86 (0.69–1.14)
BUN (mg/dL): median (IQR)	2359 (1.9%)	17.4 (12.0–26.6)	17.4 (12.0–26.6)
Lactate (mmol L^−1^): median (IQR)	46275 (36.4%)	1.5 (1.1–2.5)	1.5 (1.0–2.4)
Albumin (g L^−1^): median (IQR)	75421 (59.3%)	35 (29–41)	34 (28–40)
Glasgow Coma Score: median (IQR)	874 (0.7%)	15 (15–15)	15 (15–15)
CT scan performed: *n* (%)	1268 (1.0%)	87,294 (85.9%)	20,037 (86.3%)
Peritoneal soiling: mode (*n*, %)	399 (0.3%)	None (39,946, 39.0%)	None (9277, 39.7%)
Malignancy: mode (*n*, %)	381 (0.3%)	None (79,613, 77.8%)	None (18,255, 78.0%)
Indication: mode (*n*, %)	28,707 (22.6%)	Small bowel obstruction (18,777, 23.6%)	Small bowel obstruction (4346, 24.2%)

*BUN* blood urea nitrogen, *COPD* chronic obstructive pulmonary disease, *CT* computed tomography, *IQR* interquartile ranges.

^a^All variables apart from sex are used as covariates in RUNE.

^b^Number (%) of missing values is quoted for all cases, before excluding those with missing values for the NELA Calculator covariates.

^c^Characteristics are listed for the development and validation cases in the first cross-validation split. These development cases were used during the manual development of RUNE, and include cases with missing values. The validation cases exclude cases with missing values for the NELA calculator covariates. Percentages are calculated excluding any missing values, i.e., the denominator is the total number of non-missing values for that variable.

Variables except lactate and albumin had few missing values aside from that induced by our preprocessing of the surgical indication variable. 27.2% of cases were incomplete for these variables and 28 imputations were performed^14^. 71.2% of cases were incomplete for lactate or albumin, and 84 imputations were performed, equalling three imputations for each complete case created during previous imputation steps or 84 imputations for cases that were complete initially. RUNE, therefore, predicts 420 mortality risks for each case.

### Model performance

**Fig. 2:**
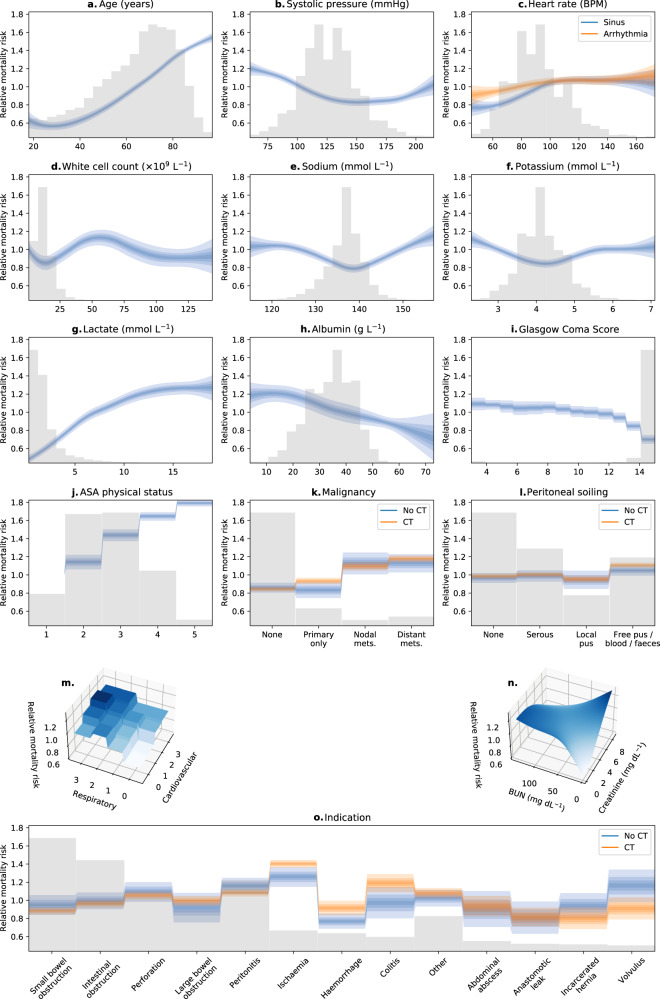
Changes in predicted mortality risk with each RUNE variable. “Partial dependence” plots in panels **a**–**o** show how the mortality risk predicted by RUNE changes with each variable if the other variables are held fixed. Blue and orange bands show changes in predicted mortality risk. In panels, **a**–**l** and **o**, the band’s width for each variable is proportional to the uncertainty of its association with mortality risk. From lightest to darkest, the four overlapping bands represent 95, 70, 45, and 20% confidence. Grey histograms show the distribution of each variable. Base categories are excluded. In panel **m**, respiratory status is encoded as 0: No dyspnoea, 1: Mild COPD or dyspnoea, 2: Moderate COPD or dyspnoea, 3: Fibrosis or consolidation or severe dyspnoea. Also in panel **m**, cardiovascular status is encoded as 0: No cardiac failure, 1: Cardiovascular medications, 2: Peripheral oedema or taking warfarin, 3: Raised jugular venous pressure or cardiomegaly.

**Fig. 3:**
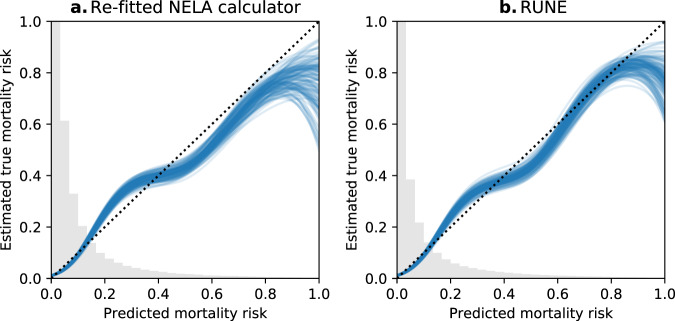
Calibration curves. Calibration curves are shown for the re-fitted NELA Calculator (**a**) and RUNE (**b)**. Each of the 120 curves for each model is derived using one of the 120 validation sets in the cross-validation. Grey histograms show the distribution of each model’s predicted mortality risks (the range of point estimates of risk across all cases) across all validation sets.

RUNE demonstrated good discrimination and calibration and improved upon the re-fitted NELA Calculator in all validation scores. Full results are shown in Table 2. Figure 2 shows the contribution of each RUNE variable to predicted mortality risk if the other variables are held fixed. Calibration curves are shown in Fig. 3. Both models rarely made very high mortality risk predictions, thus calibration for very high-risk patients could not be accurately assessed.

**Table 2:** Performance of RUNE and the re-fitted NELA calculator.

Score	Re-fitted NELA calculator^a^	RUNE^a^	Difference per development-validation split^a^
AUROC^b^	0.851 (0.842–0.859)	0.867 (0.859–0.873)	0.019 (0.016–0.022)
Tsur’s discrimination coefficient^b^	0.22 (0.208–0.235)	0.257 (0.245–0.271)	0.035 (0.029–0.041)
Log loss^c^	0.263 (0.252–0.273)	0.25 (0.24–0.261)	−0.015 (−0.017 to −0.014)
Brier score^c^	0.079 (0.075–0.082)	0.075 (0.071–0.078)	−0.005 (−0.005 to −0.004)
Mean absolute calibration error^c^	0.066 (0.047–0.089)	0.042 (0.031–0.059)	−0.026 (−0.044 to −0.004)

*NELA* National Emergency Laparotomy Audit, *AUROC* area under the receiver operating characteristic curve.

^a^Reported as median (95% confidence interval) scores across 120-fold cross-validation.

^b^Higher values indicate better performance.

^c^Lower values indicate better performance.

Uncertainty varied significantly between cases, and increased where imputation occurred: RUNE’s median (2.5th–97.5th percentile) risk distribution range was 1.6% (0.2–12.9%) for cases with no prospective imputation, 3.8% (0.3–18.3%) where only albumin was imputed, 2.3% (0.3–27.1%) where only lactate was imputed, and 2.9% (0.4–32.3%) where lactate and albumin were both imputed.

Supplementary Note E describes the performance of the albumin and lactate imputation sub-models. Supplementary Notes F and G show the outcomes of the sensitivity analyses.

### Case study

**Fig. 4:**
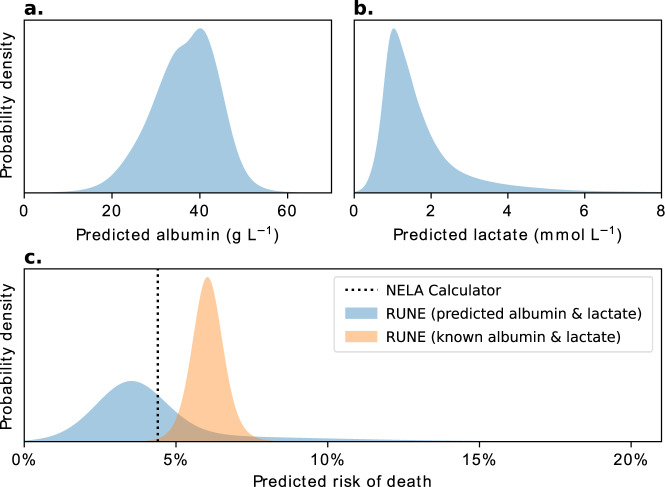
RUNE’s predictions for the case study patient. Lactate and albumin are not measured when the patient is first assessed, and our sub-models, therefore, predict distributions of plausible values for them (**a**, **b)**. Based upon this, RUNE predicts a wide mortality risk distribution (**c**, blue), indicating significant uncertainty which is masked by the point estimate from the NELA calculator. Once albumin and lactate are measured and found to be surprisingly deranged, the predicted albumin and lactate are disregarded, and RUNE makes a confident prediction of higher mortality risk (**c**, orange).

Here, we compare predictions from the NELA Calculator and RUNE for a hypothetical patient, illustrating the potential impact of uncertainty on clinical decision making. Figure 4 compares these predictions graphically.

An 81-year-old man presents to the hospital with abdominal pain and vomiting. He has essential hypertension but is usually well. Laboratory results are unremarkable except for a mildly elevated blood urea nitrogen of 24.4 mg dL^−1^. Imaging confirms small bowel obstruction and he is admitted for initial conservative management. A decision to operate is made after 48 h with no improvement, and the NELA calculator predicts a 4.4% risk of death. The patient is thus designated ‘lower risk’, where the risk of death >5% is recommended by several national bodies as defining the ‘high risk’ patient^15,16^. After an initial laparoscopic approach, he is converted to open surgery and requires a seven-day unplanned critical care admission and a further 3 weeks on the surgical floor before discharge home.

In contrast to the NELA Calculator, RUNE initially predicts a wide risk distribution consistent with a higher chance of death (see Fig. 4), reflecting the uncertainty from unmeasured albumin and lactate. When measured, the values of these variables are more extreme than expected, though still consistent with the imputation models’ predictions: albumin 25 mg dL^−1^ and lactate 3.2 mmol L^−1^. RUNE updates its predicted risk distribution to reflect this, confidently predicting a higher risk that is within the bounds of the less-confident original prediction. More realistic prognostication in this patient, supported by RUNE, could have triggered preoperative optimisation by the medical liaison team, direct supervision by senior clinicians (consultant anaesthetists, intensivists and surgeons) and planned postoperative critical care admission.

## Discussion

Our methods highlight uncertainty in the output from clinical prediction models. RUNE quantifies uncertainty over the risk of death for individuals undergoing EL and was rigorously developed and validated using data from 127,134 emergency laparotomies, a significantly larger patient cohort than previous studies. Our model builds upon the successes of the NELA calculator, captures plausible relationships between clinical variables and mortality, and is straightforward to interpret. The risk distributions it displays have the potential to inform clinical decision-making and assist discussions with patients and their families. An online calculator and application programming interface (API) for the production version of RUNE is available at laparotomy-risk.com.

Our method for uncertainty quantification generalises to prediction of other outcomes in other patient cohorts. Our robust analysis code was developed in accordance with best practice guidelines^17^, and is publicly available under MIT License at https://github.com/finncatling/lap-risk. This codebase is a significant contribution of our work, allowing not only for reproduction of our results but also for easy adaptation of our methods to other sources of data.

RUNE performed consistently well in cross-validation, despite variations in data between different hospitals. This consistency across a large number of train-test splits provides strong assurance that the production version of RUNE will perform similarly well.

RUNE quantifies two forms of uncertainty. Firstly, the dispersion of the posterior distribution over RUNE’s coefficients corresponds to imprecision (i.e. random error) in its average mortality risk estimate for a group of patients with similar values of the input variables. This first form of uncertainty could be summarised as a *credible interval* for mortality risk, and in patients where albumin and lactate are known, their risk distribution range is the 95% credible interval. The average mortality risk estimate may be biased (i.e. subject to systematic error) in patient cohorts who are unrepresentative of the NELA cohort. Such bias might occur where the values of unmeasured determinants of mortality risk change over time and across healthcare systems.

Secondly, prospective imputation of missing albumin and lactate results in a ‘personalised’ increase in uncertainty over predicted mortality risk. This corresponds to the expansion of the credible interval to approximate the *prediction interval* for mortality risk. Our method for approximating the prediction interval is well suited to variables which are measured in some cases and unmeasured in others. However, it does not extend to determinants of mortality risk which are never measured. Therefore, RUNE’s predicted distributions do not quantify the uncertainty arising from these. More generally, RUNE quantifies uncertainty within the confines of its specified mortality risk model, but does not consider uncertainty over the model specification, e.g. by considering competing plausible models with different covariates or interactions.

Figure 2 shows plausible relationships between RUNE’s variables and mortality risk. In common with the previous models^8,18^, the American Society of Anesthesiologists (ASA) physical status was strongly associated with mortality risk. This may be because, as well as encoding past medical history, ASA physical status captures a subjective clinical assessment that is otherwise missing from RUNE.

Lactate and albumin were strongly associated with mortality risk, and add useful information to RUNE that is not contained in its other covariates. These variables may not be measured in the early part of a patient’s admission when informed discussion of risk will nonetheless be desired. By allowing albumin and lactate to be missing when these initial mortality risk predictions are made, our approach enables these important variables to inform care earlier in the patient’s journey.

Precise assessment of intra-abdominal pathology is challenging in the absence of cross-sectional imaging, and accordingly, RUNE predicts higher mortality risk when suspicion of severe peritoneal soiling or ischaemic bowel is supported by a CT scan. Similarly, RUNE’s predicted risk rises steadily with the severity of CT-confirmed malignancy, but rises in a binary fashion when CT is not performed, reflecting less-precise assessment in the latter case.

Given the interdependency of the variables, each plot in Fig. 2 must be interpreted in the context of the others. For example, it is implausible that a combination of very high creatinine and urea confers an overall reduced mortality risk. Rather, patients with very high creatinine and urea are likely to have derangements in other RUNE variables which capture their mortality risk instead.

30-day mortality after emergency laparotomy in England and Wales fell from 11.8% in 2013–2014 to 9.3% in 2018–2019^6^. We were unable to model changes in mortality risk over time, or to restrict model validation to more recent cases, as dates were redacted in the dataset provided for our study. We were unable to link our data to other sources, thus our mortality outcome does not capture patient deaths occurring after hospital discharge but within 60 days of surgery. However, previous studies have noted high concordance between deaths recorded in the NELA and by the UK’s Office of National Statistics^8^. This difference in mortality outcome may also have influenced the performance of the re-fitted NELA Calculator, as it was originally designed to predict the risk of 30-day all-cause mortality.

Patients are only recorded in the NELA if they have surgery. In a recent study^19^, EL was deemed inappropriate in more than 30% of patients after they were initially considered for surgery. Caution should be exercised when using RUNE (or the NELA calculator) to inform the care of this patient population, as it is not validated in them. More generally, predictive models can reinforce bias^20^ if they are used to deny treatments to particular groups, and data from those groups are censored as a consequence.

Graphics can improve understanding of risk^21^, and probability density plots in particular are a powerful tool for communicating uncertainty^22^. Despite this promise, further work is needed to prospectively assess how predicted risk distributions will inform discussions and decision making in clinical practice. Qualitative research in this area has the potential to improve our understanding and enhance the translation of risk models to the bedside.

Survival is not the only thing that matters to patients. In future studies, we plan to extend RUNE to predict other outcomes with important implications for quality of life^23^, e.g. discharge to a patient’s own home versus to a residential care or nursing home. Estimating the risk of specific complications, such as an anastomotic leak, may also inform care.

Our methods highlight the uncertainty in the output from clinical prediction models and allow early prognostication in patients where some variables are not yet measured. RUNE builds upon the successes of the NELA calculator by improving mortality risk prediction for patients undergoing emergency laparotomy and has the potential to inform decision-makers and assist discussions with patients and their families. Our analysis code was robustly developed and is publicly available for easy adaptation to predicting other outcomes.

## Methods

The study was prospectively approved by the Imperial College Research Ethics Committee (ref:18IC4727) on 29 August 2018. The source data were collected for the purpose of a national clinical audit under Section 251 of the National Health Service Act (2006) so individual patient consent was not required.

Data were provided by the NELA^6^ in anonymised form and were stored and analysed within the secure Big Data and Analytical Unit at Imperial College London. Data analysis was conducted with Python 3.8.6 and R 4.0.3. Model fitting used Statsmodels 0.12.0^24^, PyGAM 0.8.0^25^ and Lme4 1.1–23^26^.

### Study population

For inclusion in our study, we considered all patients entered into the NELA database since its inception on 1 December 2013, whose 60-day follow-up period had ended by 21 May 2019. This comprised adults in English and Welsh hospitals who underwent emergency surgery on the gastrointestinal tract, excluding appendicectomy and cholecystectomy. Most commonly, this surgery was EL for treatment of bowel obstruction or intra-abdominal infection. Exhaustive inclusion criteria are available from the NELA^27^.

In-hospital mortality was defined as death in hospital before day 60 after surgery. Patients who remained alive in hospital on day 60 were treated as discharged alive for modelling purposes. Patients below 18 or above 109 years of age were excluded from the study.

The unit of observation in the NELA is a surgery and associated perioperative care (referred to as a ‘case’). The NELA only records the first EL in each hospital admission, and a minority of its cases (2% in November 2015^28^) are from patients who had an EL recorded in the NELA during a previous admission. Our anonymised data lacked identifiers for such patients, and we, therefore, treated all cases independently.

### Conceptual overview

We produced RUNE: a model to predict the risk of in-hospital mortality in patients having EL. Initially, 80% of hospitals were randomly selected, and cases from these were used to remove extraneous input variables and to manually tune RUNE’s regularisation parameters. We then validated its predictive accuracy on unseen cases from the remaining 20% of hospitals, approximating its performance in prospective use where patient populations and patterns of practice may change.

To calculate confidence intervals for this performance, we used cross-validation: development-validation splitting was repeated a total of 120 times, with models re-fit on the development cases (without any variable selection or manual parameter tuning) and tested on the validation cases.

We compared RUNE with the NELA calculator. To ensure a fair comparison, we recreated the NELA calculator according to its technical specification^8^ and re-fit it using our study data. As per this specification, cases with missing data were excluded. The NELA Calculator avoids variables with a high proportion of missing values in order to limit the number of discarded incomplete cases. Thus, to allow a direct comparison of the NELA calculator and RUNE, we used data from the same cases (excluding those with missing data for the NELA calculator covariates) to test both models.

Unlike the complete-case analysis used by the NELA Calculator, RUNE handles missing data using multiple imputations. This imputation is probabilistic: rather than filling in missing data with the most-likely value, a set of plausible values are simulated using the posterior predictive distribution of the missing data given the observed values of the other variables. All variables with missing values were multiply imputed when fitting RUNE. In addition, as lactate and albumin are frequently unmeasured at the time of mortality risk estimation, we designed RUNE so that lactate and albumin can be imputed prospectively when it is used in practice.

### Developing RUNE

Preoperative variables were manually inspected and implausible values redacted as described in Supplementary Note A. Continuous variables were Winsorized at the 0.1 and 99.9% percentiles, except age which was Winsorized at the 99.9% percentile only. The categorical variable encoding ECG abnormalities was rationalised to a binary variable encoding presence of any arrhythmia. The variable encoding surgical indication was consolidated to the 13 most commonly-chosen indications, plus an ‘other indication’ category, as described in Supplementary Note A.

We selected, a priori and based on clinical plausibility, 26 variables from the NELA dataset as candidates for inclusion in RUNE. These excluded variables which are more related to quality of care than patients’ own characteristics, and variables which could only be measured during or after surgery. We envision that users will enter data manually, and so aimed to limit the number of variables in the final version of RUNE. We thus used backward elimination to exclude the seven candidate variables most weakly associated with mortality risk in multivariable modelling. Full details of this process are described in Supplementary Note B.

Candidate variables except lactate and albumin are measured routinely. Therefore, we treated them as missing at random and imputed them during model fitting only. This allowed us to include mortality as a covariate in their imputation sub-models to avoid biasing their coefficients^14^. Missing values for binary and continuous variables (apart from lactate and albumin) were multiply imputed with chained equations (MICE) as described in Supplementary Note C.

Lactate and albumin may be more likely to be measured in patients who are especially unwell. We treated them as missing at random, under the hypothesis that unwellness is closely related to mortality risk, and RUNE’s variables were selected specifically to quantify this. We tested this hypothesis by deriving lactate and albumin missingness-indicator variables for use as RUNE covariates, as explained in Supplementary Note C. Generalised additive models (GAMs)^29^ were specified for lactate and albumin imputation, and took as input the results of the MICE and the categorical imputation. Lactate and albumin were both multiply imputed by sampling from the posterior distributions over their respective imputation model coefficients.

RUNE is also a GAM (in this case, with a binomial error distribution and a logit link^25^). In all GAMs, continuous variables were transformed using penalised B splines, with 10 second-degree polynomial splines per variable and linearly-spaced knots. Discrete variables were encoded with the base category excluded to avoid the dummy variable trap^30^. The smoothness of each spline term was controlled via a penalty on its second derivative^31^. Inter-category differences for each discrete variable were limited via a penalty on its L2 norm^25^. These regularisation terms were manually tuned to obtain a clinically-plausible fit for each variable, via inspection of the partial dependence plots for GAMs fit to the initial development cases. The investigators were blinded to the downstream effects on model performance during this manual tuning, and tuning was finalised prior to model validation.

We specified tensor-product interactions^31^ between heart rate and the presence of arrhythmia, between blood urea nitrogen and creatinine, and between cardiovascular and respiratory status. Clinicians entering data into the NELA are asked to predict abdominal pathology that will only be confirmed intra- or postoperatively. Cross-sectional imaging may make these estimates more reliable. In order to capture this, we specified tensor-product interactions between whether preoperative computed tomography (CT) abdomen and pelvis was performed and each of: predicted degree of peritoneal soiling, presence of malignancy and surgical indication.

Given a case with missing data, our multiple imputation process yields several complete versions of the case. Thus, imputation for the development cases yielded multiple complete datasets. We fitted a GAM on each of these datasets and combined the GAMs using Rubin’s rules, producing a single GAM with robust coefficients that account for uncertainty due to missing data^14^. This process is described fully in Supplementary Note C.

Each round of cross-validation included re-Winsorization, re-fitting of imputation sub-models, multiple imputations and re-fitting RUNE.

Mortality risk distributions were predicted for each case as follows: Missing data were multiply imputed, then five predicted risks were obtained for each of the many complete versions of the case by sampling from the approximate posterior distribution over RUNE’s coefficients^31^. The set of predicted mortality risks for each case can be displayed either as a histogram or transformed into a risk distribution via kernel density estimation.

### Model validation and comparison

We report the median performance of each model in cross-validation, and use the 2.5th and 97.5th performance percentiles as a 95% confidence interval. The area under the receiver operating characteristic curve, log loss, Brier score and Tjur’s discrimination coeffecient^32^ are reported. We also generate smooth calibration curves as described in Supplementary Note D and report calibration error as the mean absolute error between each curve and the line of identity. For the re-fitted NELA Calculator, these scores are calculated using the single point prediction of mortality risk it generates for each case. For RUNE, we calculate the means of these scores across each of the multiple risks it predicts per case.

To measure the uncertainty around risk predictions for individual patients, we calculate the range between the 2.5th and 97.5th percentiles (termed the *risk distribution range*) for each risk distribution predicted in the initial validation cases. We group the risk distribution ranges together according to whether lactate or albumin were imputed, then report the median, 2.5th and 97.5th percentiles for each group.

Following validation, a production version of RUNE was re-fitted using all the study data.

### Sensitivity analyses

We aimed to allow prospective use of RUNE in patients where lactate and albumin were not yet measured. To this end, we excluded mortality as a covariate in the lactate and albumin imputation sub-models. We analysed the sensitivity of RUNE’s lactate and albumin spline terms to this exclusion.

Albumin measurements were not recorded in the NELA until December 2016, meaning that their missingness changes over time. Dates were redacted in the dataset provided for our study, meaning that we were unable to model temporal trends during albumin imputation. We thus analysed the sensitivity of RUNE’s performance by simply excluding albumin as a covariate.

### Reporting summary

Further information on research design is available in the Nature Research Reporting Summary linked to this article.

## Supplementary information

Supplement

Reporting Summary Checklist

## Data Availability

Under the terms of the data-sharing agreement for this study, we are unable to share the source data directly. Requests for anonymous patient-level data can be made directly to the NELA Project Team.

## Code availability

Our analysis code is publicly available under MIT License at https://github.com/finncatling/lap-risk.
