# Supplementary material for: Highlighting uncertainty in clinical risk prediction using a model of emergency laparotomy mortality risk
Source: NPJ Digit Med. 2022 Jun 8;5:70. doi: 10.1038/s41746-022-00616-7 (PMC9177766; doi:10.1038/s41746-022-00616-7)
Supplement: Supplementary file 1 — Supplement [file 41746_2022_616_MOESM1_ESM.pdf]

Supplementary material for *Highlighting uncertainty in clinical risk prediction using a model of emergency laparotomy mortality risk*

Contents

Supplementary Note A: Data preparation
Supplementary Note B: Selection from RUNE candidate covariates
Supplementary Note C: Missing data imputation
Supplementary Note D: Evaluating model calibration
Supplementary Note E: Performance of the albumin and lactate imputation sub-models
Supplementary Note F: Sensitivity to mortality covariate in lactate and albumin imputation models
Supplementary Note G: Sensitivity to excluding albumin as a RUNE covariate

Supplementary Table 1. Redaction criteria for implausible values of preoperative variables
Supplementary Table 2. Steps taken to consolidate surgical indications
Supplementary Table 3. Performance of the 'no albumin' version of RUNE

Supplementary Figure 1. Preoperative creatinine and BUN before and after removal of implausible values
Supplementary Figure 2. Partial dependence plots for albumin imputation sub-model
Supplementary Figure 3. Partial dependence plots for lactate imputation sub-model
Supplementary Figure 4: Sensitivity of lactate and albumin spline terms in RUNE
Supplementary Figure 5. Partial dependence plots for the 'no albumin' version of RUNE

Supplementary References

Supplementary Note A: Data preparation

Implausible values for continuous preoperative variables were redacted as shown in Supplementary Table 1. Bivariate scatterplots of preoperative blood urea nitrogen (BUN) and creatinine values suggested that, in a small minority of cases, creatinine values had erroneously been recorded as BUN values and vice versa. We addressed this by redacting both BUN and creatinine values for 222 cases where these values were the same, and for 199 cases where BUN was greater than 84 mg dL^{-1} and also greater than 2 × creatinine. Supplementary Figure 1 shows the effect of this policy.

The NELA data collection proforma allows users to select one or more of 27 surgical indications, some of which are rarely chosen. Whilst ≤2 indications are selected for most cases, a minority have a large number of chosen indications. Including all indications in RUNE, in any combination, would be overwhelming for users and create many rarely-used categories. We therefore opted to consolidate the indications to only those that were chosen most commonly, and to limit each case to one indication.

Common single indications were defined as those chosen solely in ≥1200 of cases. The most commonly-chosen pairs and trios of indications were manually consolidated to the most similar common single indication. Remaining cases were defined as 'Other indication' or as missing. Missing indications were multiply imputed as described in the main manuscript. Full details of the indication consolidation process are shown in Supplementary Table 2.

Supplementary Note B: Selection from RUNE candidate covariates

The 26 candidate covariates for RUNE were: age, sex, American Society of Anesthesiologists (ASA) physical status, cardiovascular status, respiratory status, Glasgow coma score, whether CT scan had been performed, predicted degree of peritoneal soiling, presence of malignancy, surgical indication, surgical urgency, number of surgeries within 30 days, severity of the intended surgery, predicted blood loss, heart rate, presence of arrhythmia, systolic arterial pressure, white cell count, haemoglobin, C-reactive protein, sodium, potassium, creatinine, blood urea nitrogen (BUN), lactate and albumin.

Missing values were imputed as described in the main manuscript and Supplementary Note C, except that lactate and albumin were imputed in the same way as the other continuous variables (linear regression followed by predictive mean matching) rather than using customised GAMs. To limit the computational intensity of the analysis, which was increased by the cross validation described below, we performed ten rounds of multiple imputation rather than the larger number used in the main analysis.

Several variables (heart rate, presence of arrhythmia, BUN, creatinine, cardiovascular status, respiratory status, whether CT scan had been performed, predicted degree of peritoneal soiling, presence of malignancy, surgical indication) were used in prespecified tensor-product interaction terms. These terms were considered *a priori* important and their constituent variables were therefore not considered for backward elimination.

Seven of the remaining 16 candidate covariates were removed via backward elimination[1]. This process used only the development data from the first development-validation split. During this process, the mutual information of the covariates may result in weak association with mortality risk for some covariates despite a strong association with mortality risk in univariable modelling.

A version of RUNE containing all the candidate covariates (referred to as *full RUNE*) was specified, and its regularisation terms were tuned, as described in the main manuscript. In each of the seven rounds of backward elimination, each of the remaining candidate covariates was considered for elimination as follows:

1. Remove the candidate covariate under consideration (plus any previously-eliminated covariates) from full RUNE, yielding *candidate RUNE*.
2. Split each of the ten datasets from multiple imputation into train and test subsets, in a 2:1 ratio.
3. Using the ten train subsets, fit ten versions of candidate RUNE then combine them using Rubin's rules as described in Supplementary Note C, yielding *combined candidate RUNE*.
4. Calculate the log loss of combined candidate RUNE on each of the ten test subsets.
5. Repeat steps 2-4 twice more, i.e. perform three-fold cross validation overall.
6. Calculate the mean of the 30 log losses obtained overall.

The candidate covariate which yields the lowest mean log loss (i.e. which diminishes predictive performance the least) in that round is then eliminated.

Supplementary Note C: Missing data imputation

The different imputation models used in our analysis necessitated that two rounds of multiple imputation were conducted in series whilst fitting RUNE: In the first round, variables apart from lactate and albumin were imputed, whilst lactate and albumin alone were imputed in the second. The minimum number of imputations required for the jth round was determined as m_j = ceiling($f_j \times 100$), where f_j is the fraction of cases that are incomplete for any variable being imputed in round $j \in \{1, 2\}$. In general, f_j is at least as large as the fraction of missing information[2]. m_1 imputations were performed in round one. The number of imputations round two was the smallest integer greater than or equal to m_2, that was also a multiple of m_1.

MICE for binary and continuous variables (apart from lactate and albumin) used logistic and linear regression respectively, without transformation of the imputation model covariates, followed by *predictive mean matching*. In predictive mean matching, the output of the regression model is compared to the observed values for the variable being imputed, and its 20 nearest observed neighbours (by Euclidean distance) are found. The imputed value is sampled from a discrete uniform distribution over these nearest neighbours.

Different imputed values were obtained at each MICE iteration by setting the imputation models' coefficients to samples from their approximate posterior distributions. For each categorical variable, a categorical distribution was obtained using multinomial regression on the MICE output (again, without transformation of the imputation model covariates), then the variable was multiple imputed by repeated sampling from this distribution.

To validate our treatment of lactate and albumin as missing at random, we derived lactate and albumin missingness-indicator variables for use as RUNE covariates. We hypothesised that, if lactate or albumin were more likely to be missing in patients who were less unwell, and the other RUNE covariates failed to fully quantify this wellness, then the missingness-indicator variables would be significantly associated with mortality risk. After fitting RUNE as described in the main manuscript, we calculated p-values for the missingness indicator terms. The null hypothesis for each term was that its coefficient was zero, after compensating for bias in the other model terms[3]. P-values (1 degree of freedom) for both missingness indicators were 1.0 in every cross-validation fold, validating our treatment of lactate and albumin as missing at random.

GAMs (for lactate and albumin imputation, and RUNE itself) fit on the complete datasets output from multiple imputation were combined using Rubin's rules[2]. Specifically, given m complete datasets from multiple imputation, we fit m GAMs with approximate posterior distributions $\{N(\mu_i, \Sigma_i) : 1 \leq i \leq m\}$ over their coefficients. The combined GAM has coefficients with mean $\mu^* = \frac{1}{m}\sum_{i=1}^{m} \mu_i$ and covariance $\Sigma^* = \left(1 + \frac{1}{m}\right) B + \frac{1}{m}\sum_{i=1}^{m} \Sigma_i$ where $B = \frac{1}{m-1}\sum_{i=1}^{m}(\mu_i - \mu^*)(\mu_i - \mu^*)^T$.

Supplementary Note D: Evaluating model calibration

We obtained smooth calibration curves for the re-fitted NELA Calculator and RUNE as follows: Given the validation-set mortality labels $\boldsymbol{y}$ and corresponding predicted mortality risks $\hat{\boldsymbol{y}}$, we use a GAM to model $y_i \sim$ Bernoulli(p=σ(f($\hat{y}_i$) + intercept)), where f is a spline transformation using 5 second-degree polynomial splines with a smoothing penalty on their second derivative, and σ is the logistic function. Given a predicted mortality risk, this GAM thereby estimates the corresponding true mortality risk. We fit a separate calibration GAM on each of the 120 validation sets, and obtain a smooth calibration curve for each by evaluating the fitted GAM over the interval [0, 1].

For the re-fitted NELA Calculator, we choose the smoothing penalty which produced the best fit amongst 50 candidates logarithmically spaced between 10^{-3} and 10^{3}. The calibration GAM fitting for RUNE was too computationally intensive (as the fitting process uses 420 predicted risks for each case in the validation set ≈ 10,000,000 risks) to repeat for each of 50 candidate smoothing penalties, so a two-stage smoothing penalty selection process was adopted: we derived point predictions of mortality risk from RUNE by taking the median of each predicted risk distribution, and fit calibration GAMs on these using the 50 candidate smoothing penalties described above. We found the three smoothing penalties most commonly selected in this process, and used them as candidates in the final calibration GAM fitting for RUNE.

Supplementary Note E: Performance of the albumin and lactate imputation sub-models

The imputation sub-models explained only a limited proportion of the observed variance in albumin and lactate. In the main-analysis imputation models which excluded mortality as a covariate, median (95% CI) R^2 was 0.22 (0.19 - 0.25) for the albumin imputation model and 0.22 (0.20 - 0.25) for the lactate imputation model. In the sensitivity-analysis imputation models which included mortality as a covariate, median (95% CI) R^2 was 0.23 (0.20 - 0.25) for the albumin imputation model and 0.24 (0.22 - 0.27) for the lactate imputation model. Partial dependence plots for each input variable in the main-analysis imputation models are shown in Supplementary Figures 2 and 3.

Supplementary Note F: Sensitivity to mortality covariate in lactate and albumin imputation models

As shown in Supplementary Figure 4, excluding mortality as a covariate in the lactate and albumin imputation models caused RUNE to underestimate lactate and albumin's association with mortality risk. This effect was more marked for albumin, which may result from its higher proportion of missing values.

Supplementary Note G: Sensitivity to excluding albumin as a RUNE covariate

Supplementary Table 3 compares the performance of RUNE with the sensitivity analysis version of RUNE that excludes albumin as a covariate (‘No-albumin RUNE’). Excluding albumin produced a small decrease in model discrimination with no discernible change in calibration. This reduction in predictive performance, in comparison to the main analysis, supports our including albumin as a RUNE covariate despite not being able to model its changing missingness over time.

Supplementary Figure 5 shows, for no-albumin RUNE, the contribution of each variable to predicted mortality risk if the other variables are held fixed. The relationships of the remaining covariates with mortality risk are unchanged compared with those observed in Figure 2 of the main manuscript. This supports our not including interaction terms between albumin and the other covariates in RUNE.

Supplementary Table 1. Redaction criteria for implausible values of preoperative variables

Variable	Threshold	Number (%) of cases (n=127134)
Creatinine (mg dL^{-1})	Redact if < 0.10	451 (0.355)
Blood urea nitrogen (mg dL^{-1})	Redact if > 201.7	377 (0.297)
Lactate (mmol L^{-1})	Redact if 0	28 (0.022)
Lowest albumin (g L^{-1})	Redact if 0	2 (0.002)
Systolic blood pressure (mmHg)	Redact if < 60	299 (0.235)
Heart rate (beats min^{-1})	Redact if < 30	57 (0.045)

Supplementary Table 2. Steps taken to consolidate surgical indications

**Step**	**Indication names**	**Consolidated to**	**Number (%) of cases (n=127134)**
Common single indications	Small bowel obstruction<br>Intestinal obstruction<br>Perforation<br>Large bowel obstruction<br>Peritonitis<br>Ischaemia<br>Haemorrhage<br>Colitis<br>Abdominal abscess<br>Anastomotic leak<br>Incarcerated hernia<br>Volvulus<br>Other indication	n/a	81997 (64.50)
Pairs	Perforation<br>Peritonitis	Peritonitis	8293 (6.52)
	Small bowel obstruction<br>Incarcerated hernia	Small bowel obstruction	1371 (1.08)
	Perforation<br>Abdominal abscess	Perforation	894 (0.70)
Trios	Perforation<br>Peritonitis<br>Abdominal abscess	Peritonitis	971 (0.76)
No indication	n/a	Indication missing	42 (0.03)
≥2 indications, at least one of which is a common single indication, and case not reassigned above		Indication missing	28,665 (22.55)
≥1 indication, none of which are common single indications		Other indication	4901 (3.85)

Supplementary Table 3. Performance of RUNE and the sensitivity analysis version of RUNE that excludes albumin as a covariate (‘no-albumin RUNE’)

**Score**	**RUNE**[a]	**No-albumin RUNE**[a]	**Difference per development-validation split**[a]
AUROC[b]	0.867<br>(0.859 - 0.873)	0.866<br>(0.857 - 0.872)	-0.0012<br>(-0.0017 - -0.0006)
Tsur’s discrimination coefficient[b]	0.257<br>(0.245 - 0.271)	0.255<br>(0.243 - 0.269)	-0.0018<br>(-0.0029 - -0.0009)
Log loss[c]	0.25<br>(0.24 - 0.261)	0.25<br>(0.24 - 0.262)	0.0007<br>(0.0003 - 0.001)
Brier score[c]	0.075<br>(0.071 - 0.078)	0.075<br>(0.071 - 0.078)	0.0001<br>(0.0 - 0.0002)
Mean absolute calibration error[c]	0.042<br>(0.031 - 0.059)	0.043<br>(0.031 - 0.058)	-0.0003<br>(-0.002 - 0.0014)

Abbreviation: NELA, National Emergency Laparotomy Audit; AUROC, area under the receiver operating characteristic curve

[a]Reported as median (95% confidence interval) scores across 120-fold cross validation

[b]Higher values indicate better performance

[c]Lower values indicate better performance

Supplementary Figure 1. Preoperative creatinine and blood urea nitrogen (BUN) before and after removal of implausible values.

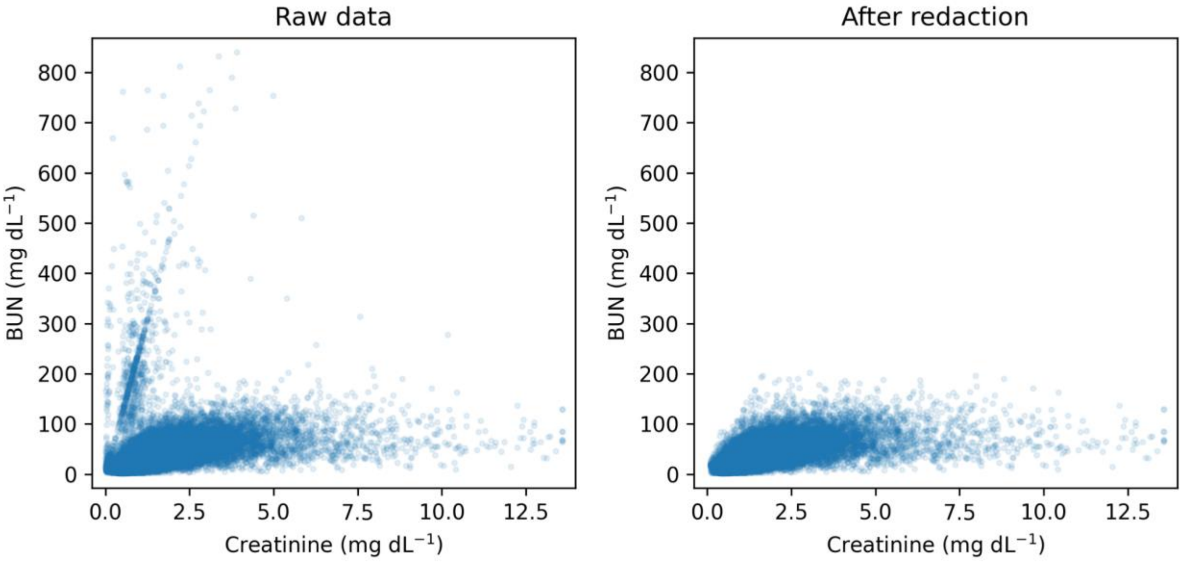

Supplementary Figure 2. ‘Partial dependence’ plots, showing how imputed albumin changes with each variable, if all other variables are held fixed. Blue and orange bands show changes in imputed albumin. The band’s width represents 95% confidence, and is proportional to the uncertainty of the association between that variable and albumin. Respiratory status is encoded as 0: No dyspnoea, 1: Mild COPD or dyspnoea, 2: Moderate COPD or dyspnoea, 3: Fibrosis or consolidation or severe dyspnoea. Cardiovascular status is encoded as 0: No cardiac failure, 1: Cardiovascular medications, 2: Peripheral oedema or taking warfarin, 3: Raised jugular venous pressure or cardiomegaly.

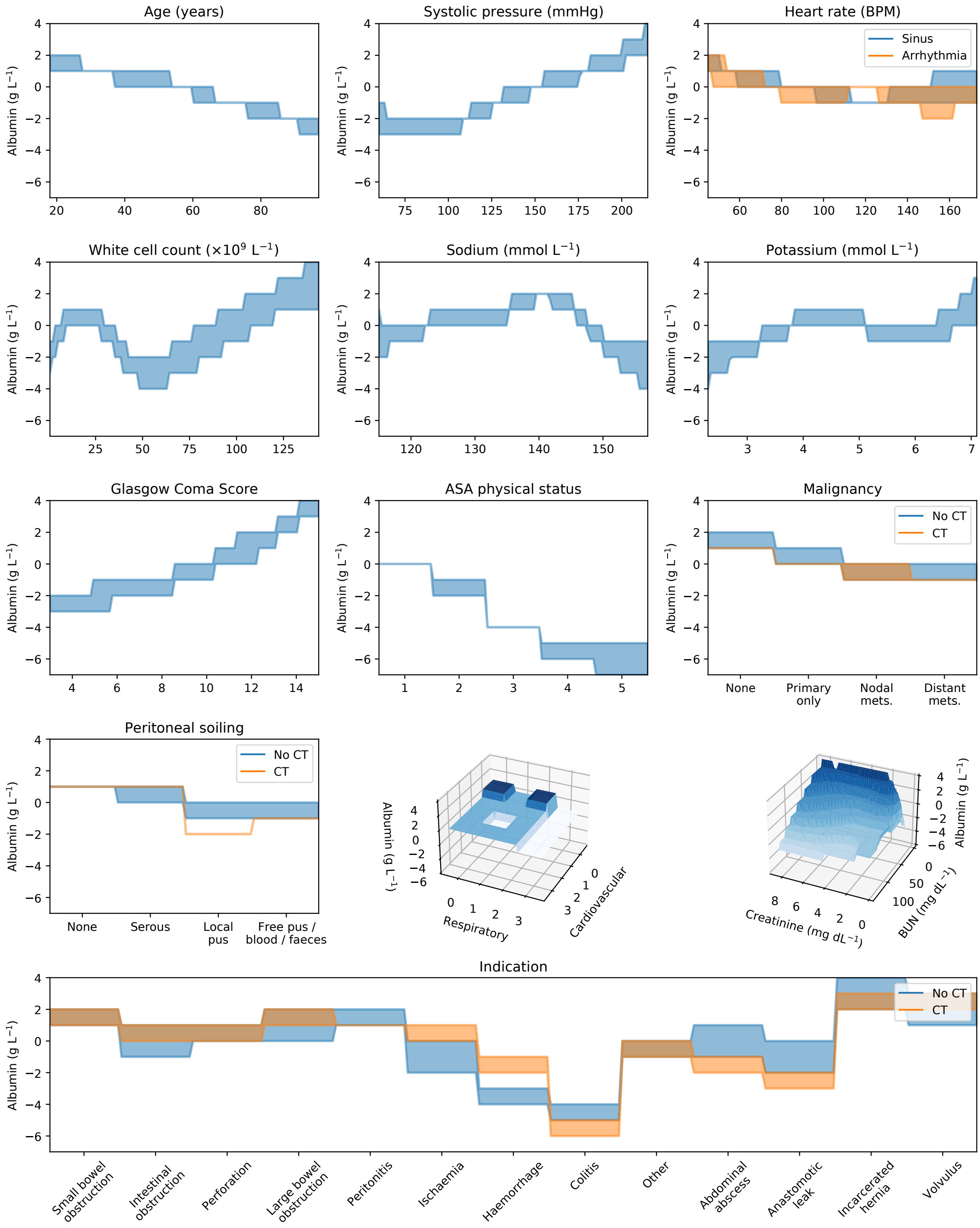

Supplementary Figure 3. ‘Partial dependence’ plots, showing how imputed lactate changes with each variable, if all other variables are held fixed. Blue and orange bands show changes in imputed lactate. The band’s width represents 95% confidence, and is proportional to the uncertainty of the association between that variable and lactate. Respiratory status is encoded as 0: No dyspnoea, 1: Mild COPD or dyspnoea, 2: Moderate COPD or dyspnoea, 3: Fibrosis or consolidation or severe dyspnoea. Cardiovascular status is encoded as 0: No cardiac failure, 1: Cardiovascular medications, 2: Peripheral oedema or taking warfarin, 3: Raised jugular venous pressure or cardiomegaly.

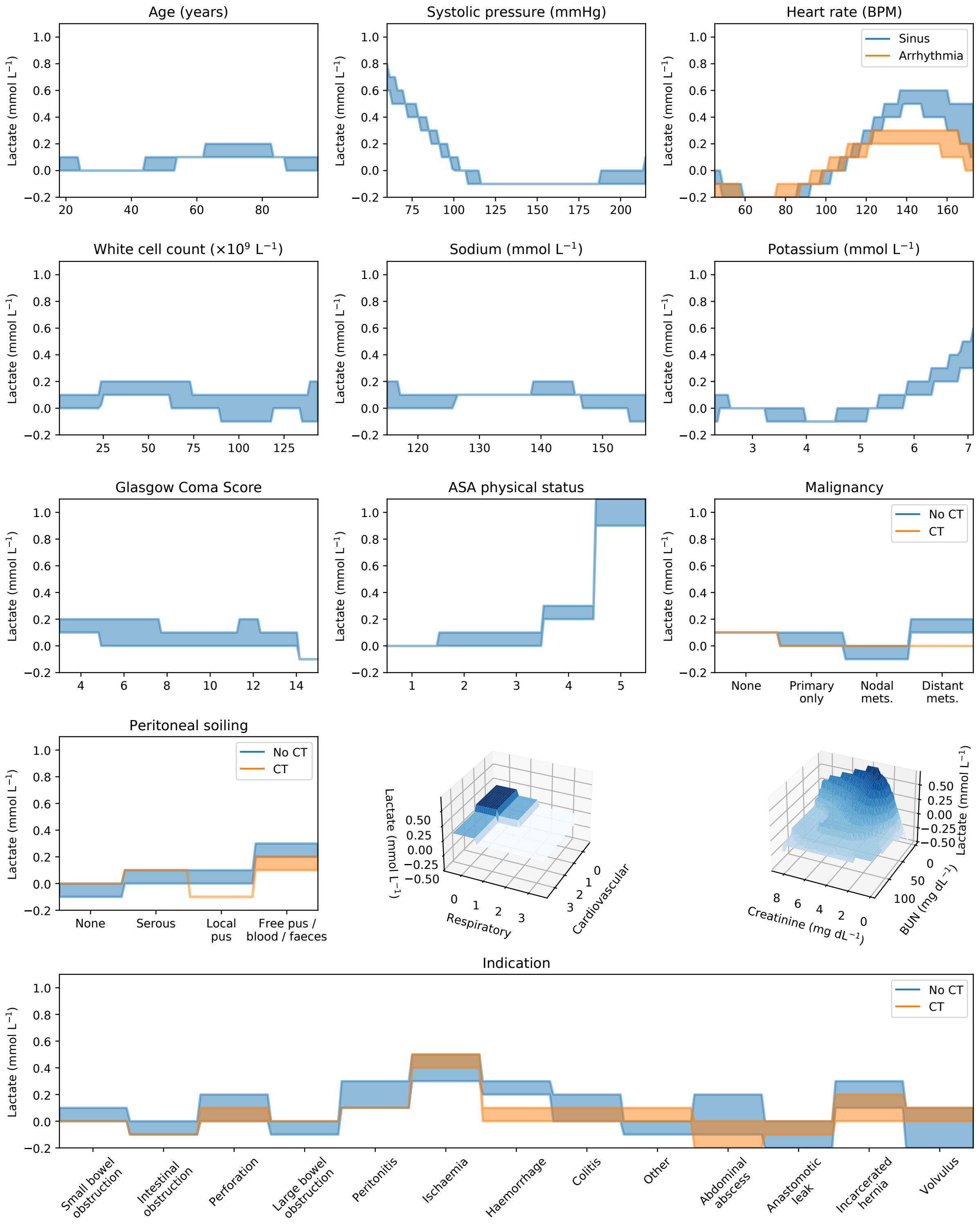

Supplementary Figure 4. Spline terms for lactate and albumin in the novel mortality risk model, both for the original (main analysis) version of RUNE which excludes mortality as a covariate in lactate and albumin imputation sub-models, and the re-fitted (sensitivity analysis) version of RUNE which includes mortality as a covariate in lactate and albumin imputation sub-models. Blue and orange bands show changes in predicted mortality risk. The band's width for each variable is proportional to the uncertainty of its association with mortality risk. From lightest to darkest, the 4 overlapping bands represent 95%, 70%, 45% and 20% confidence.

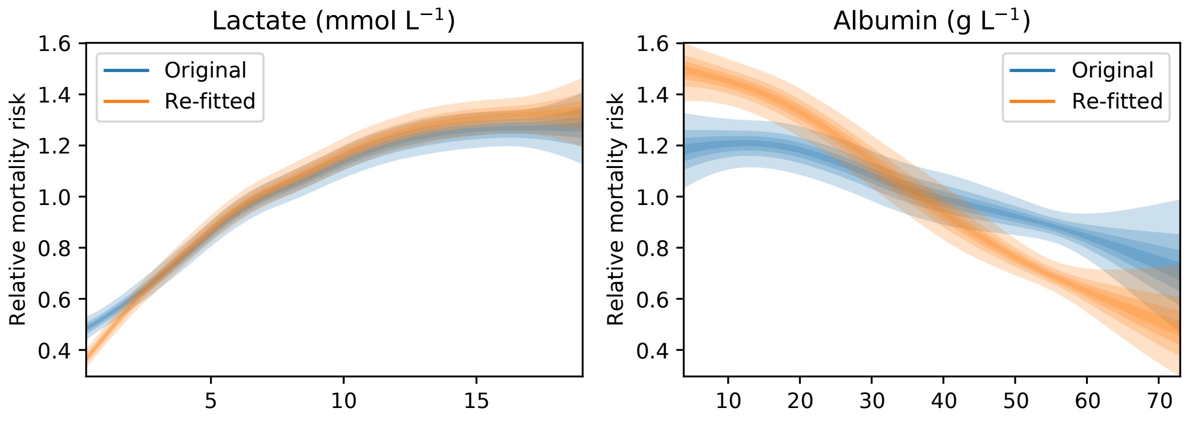

Supplementary Figure 5. 'Partial dependence' plots, showing how the mortality risk predicted by no-albumin RUNE changes with each variable, if the other variables are held fixed. Blue and orange bands show changes in predicted mortality risk. The band's width for each variable is proportional to the uncertainty of its association with mortality risk. From lightest to darkest, the 4 overlapping bands represent 95%, 70%, 45% and 20% confidence. Grey histograms show the distribution of each variable. Base categories are excluded. Respiratory status is encoded as 0: No dyspnoea, 1: Mild COPD or dyspnoea, 2: Moderate COPD or dyspnoea, 3: Fibrosis or consolidation or severe dyspnoea. Cardiovascular status is encoded as 0: No cardiac failure, 1: Cardiovascular medications, 2: Peripheral oedema or taking warfarin, 3: Raised jugular venous pressure or cardiomegaly.

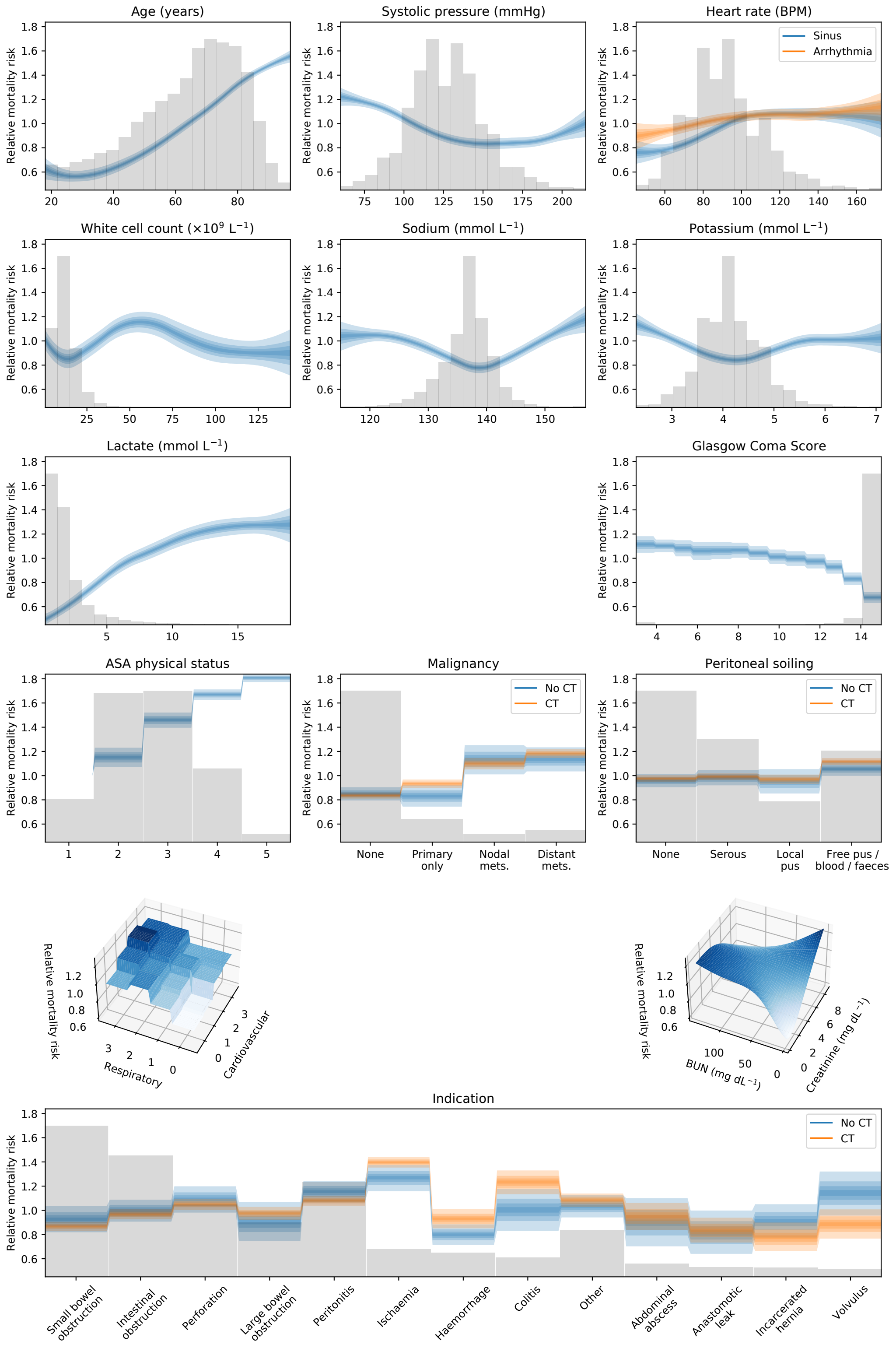

Supplementary References

1. Guyon, I., Weston, J., Barnhill, S. & Vapnik, V. Gene Selection for Cancer Classification using Support Vector Machines. *Mach. Learn.* **46**, 389–422 (2002).
2. White, I. R., Royston, P. & Wood, A. M. Multiple imputation using chained equations: Issues and guidance for practice. *Stat. Med.* **30**, 377–399 (2011).
3. Wood, S. N. *Generalized additive models : an introduction with R*. (Boca Raton, Florida : Chapman & Hall/CRC, 2006).
