# Supplementary material for: Highlighting uncertainty in clinical risk prediction using a model of emergency laparotomy mortality risk
Source: NPJ Digit Med. 2022 Jun 8;5:70. doi: 10.1038/s41746-022-00616-7 (PMC9177766; doi:10.1038/s41746-022-00616-7)
Supplement: Supplementary file 2 — Reporting Summary Checklist [file 41746_2022_616_MOESM2_ESM.pdf]

nature portfolio

Corresponding author(s): Finneas catling

Last updated by author(s): Apr 28, 2022

Reporting Summary

Nature Portfolio wishes to improve the reproducibility of the work that we publish. This form provides structure for consistency and transparency in reporting. For further information on Nature Portfolio policies, see our Editorial Policies and the Editorial Policy Checklist.

Statistics

For all statistical analyses, confirm that the following items are present in the figure legend, table legend, main text, or Methods section.

n/a	Confirmed	
☐	☒	The exact sample size (n) for each experimental group/condition, given as a discrete number and unit of measurement
☒	☐	A statement on whether measurements were taken from distinct samples or whether the same sample was measured repeatedly
☐	☒	The statistical test(s) used AND whether they are one- or two-sided<br>*Only common tests should be described solely by name; describe more complex techniques in the Methods section.*
☐	☒	A description of all covariates tested
☒	☐	A description of any assumptions or corrections, such as tests of normality and adjustment for multiple comparisons
☐	☒	A full description of the statistical parameters including central tendency (e.g. means) or other basic estimates (e.g. regression coefficient) AND variation (e.g. standard deviation) or associated estimates of uncertainty (e.g. confidence intervals)
☐	☒	For null hypothesis testing, the test statistic (e.g. F, t, r) with confidence intervals, effect sizes, degrees of freedom and P value noted<br>*Give P values as exact values whenever suitable.*
☒	☐	For Bayesian analysis, information on the choice of priors and Markov chain Monte Carlo settings
☒	☐	For hierarchical and complex designs, identification of the appropriate level for tests and full reporting of outcomes
☒	☐	Estimates of effect sizes (e.g. Cohen's d, Pearson's r), indicating how they were calculated

Our web collection on statistics for biologists contains articles on many of the points above.

Software and code

Policy information about availability of computer code

Data collection: Data was supplied directly by the UK's National Emergency Laparotomy Audit (NELA).

Data analysis: Our analysis code in publicly available under MIT license at https://github.com/finncatling/lap-risk

For manuscripts utilizing custom algorithms or software that are central to the research but not yet described in published literature, software must be made available to editors and reviewers. We strongly encourage code deposition in a community repository (e.g. GitHub). See the Nature Portfolio guidelines for submitting code & software for further information.

Data

Policy information about availability of data

All manuscripts must include a data availability statement. This statement should provide the following information, where applicable:

- Accession codes, unique identifiers, or web links for publicly available datasets
- A description of any restrictions on data availability
- For clinical datasets or third party data, please ensure that the statement adheres to our policy

Under the terms of the data sharing agreement for this study we are unable to share the source data directly. Requests for anonymous patient-level data can be made directly to the NELA project team.

Field-specific reporting

Please select the one below that is the best fit for your research. If you are not sure, read the appropriate sections before making your selection.

☒ Life sciences ☐ Behavioural & social sciences ☐ Ecological, evolutionary & environmental sciences

For a reference copy of the document with all sections, see nature.com/documents/nr-reporting-summary-flat.pdf

Life sciences study design

All studies must disclose on these points even when the disclosure is negative.

Sample size	No sample size calculation was made. Our study uses data from 127134 surgeries, a significantly larger number than previous studies which develop and validate models of mortality risk in emergency laparotomy.
Data exclusions	Cases where the admitting age was <18 or implausibly large (older than ever recorded in the UK) were excluded. Data where values were outside the detectable range of assays or, in the case of BUN and creatinine, highly likely to have been swapped were redacted and imputed later in the analysis.
Replication	120-fold cross-validation was performed. Each round of cross-validation included re-Winsorization, re-fitting of imputation sub-models, multiple imputation and re-fitting the mortality risk model.
Randomization	For each cross validation fold, 80% of hospitals were randomly selected and cases from them were used for model fitting. We then validated the fitted model's predictive accuracy on unseen cases from the remaining 20% of hospitals, approximating its performance in prospective use where patient populations and patterns of practice may change.
Blinding	Allocation was performed by the automated data pipeline and there was no opportunity for authors to interfere in the allocation process. The pipeline is open source and easily replicable. Initial model parameter tuning was done with respect to the regularisation parameters of the mortality risk model and authors were blinded to any downstream effect on model performance

Reporting for specific materials, systems and methods

We require information from authors about some types of materials, experimental systems and methods used in many studies. Here, indicate whether each material, system or method listed is relevant to your study. If you are not sure if a list item applies to your research, read the appropriate section before selecting a response.

Materials & experimental systems

n/a	Involved in the study	
☒	☐	Antibodies
☒	☐	Eukaryotic cell lines
☒	☐	Palaeontology and archaeology
☒	☐	Animals and other organisms
☒	☐	Human research participants
☒	☐	Clinical data
☒	☐	Dual use research of concern

Methods

n/a	Involved in the study	
☒	☐	ChIP-seq
☒	☐	Flow cytometry
☒	☐	MRI-based neuroimaging
